# Subchondral drilling for articular cartilage repair: a systematic review of translational research

**DOI:** 10.1242/dmm.034280

**Published:** 2018-06-19

**Authors:** Liang Gao, Lars K. H. Goebel, Patrick Orth, Magali Cucchiarini, Henning Madry

**Affiliations:** 1 Center of Experimental Orthopedics, Saarland University, D-66421 Homburg, Germany; 2 Department of Orthopaedic Surgery, Saarland University Medical Center, D-66421 Homburg, Germany

**Keywords:** Articular cartilage repair, Subchondral drilling, Systematic review, Translational research, Animal model

## Abstract

Articular cartilage defects may initiate osteoarthritis. Subchondral drilling, a widely applied clinical technique to treat small cartilage defects, does not yield cartilage regeneration. Various translational studies aiming to improve the outcome of drilling have been performed; however, a robust systematic analysis of its translational evidence was still lacking. Here, we performed a systematic review of the outcome of subchondral drilling for knee cartilage repair in translational animal models. A total of 12 relevant publications studying 198 animals was identified, detailed study characteristics were extracted, and methodological quality and risk of bias were analyzed. Subchondral drilling led to improved repair outcome compared with defects that were untreated or treated with abrasion arthroplasty for cartilage repair in multiple translational models. Within the 12 studies, considerable subchondral bone changes were observed, including subchondral bone cysts and intralesional osteophytes. Furthermore, extensive alterations of the subchondral bone microarchitecture appeared in a temporal pattern in small and large animal models, together with specific topographic aspects of repair. Moreover, variable technical aspects directly affected the outcomes of osteochondral repair. The data from this systematic review indicate that subchondral drilling yields improved short-term structural articular cartilage repair compared with spontaneous repair in multiple small and large animal models. These results have important implications for future investigations aimed at an enhanced translation into clinical settings for the treatment of cartilage defects, highlighting the importance of considering specific aspects of modifiable variables such as improvements in the design and reporting of preclinical studies, together with the need to better understand the underlying mechanisms of cartilage repair following subchondral drilling.

## Abstract (precis)

**Summary:** Cartilage defects initiate osteoarthritis. This is the first systematic review of translational evidence of cartilage defects in animal models treated by drilling, enhancing translation from basic science to clinical application.

## INTRODUCTION

Defects of the articular cartilage, the gliding tissue that covers the ends of articulating bones in all joints, do not heal. The past two decades have seen significant progress in the basic science of articular cartilage repair and new treatment modalities, for which varying degrees of cartilage repair have been reported, have emerged (Donaldson et al., 2015; Getgood et al., 2009). However, consistent, favourable and reproducible clinical results are still not available. Among these modalities, marrow stimulation techniques are established key first-line reconstructive options for small symptomatic articular cartilage defects (Steinwachs et al., 2008).

**Fig. 1.**
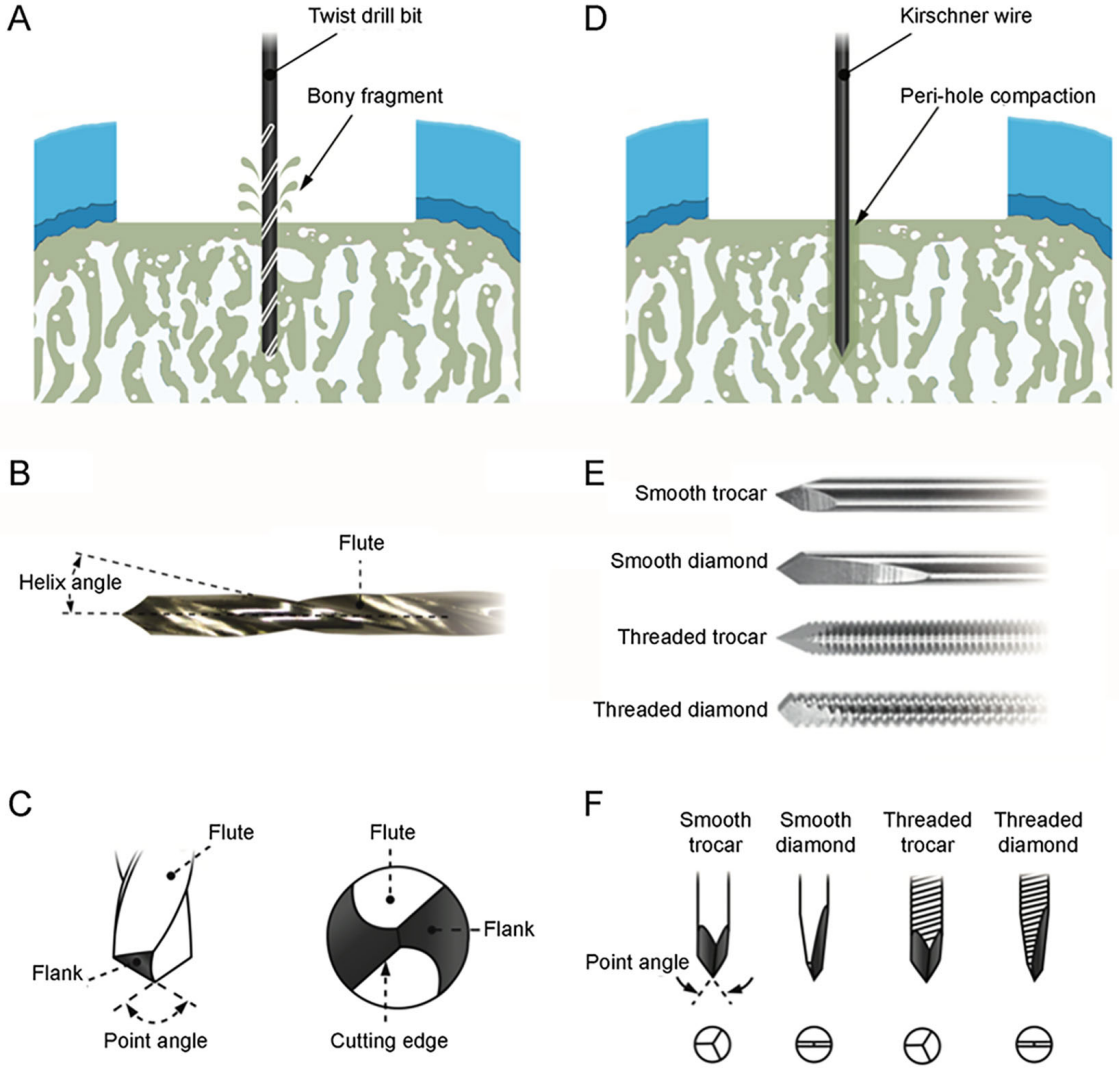
**Schematic of subchondral drilling.** Diagrams show drilling with a surgical twist drill bit (A-C) and a Kirschner wire (D-F). (A) Subchondral drilling with a standard twist drill bit. The bone fragment is released from the drilled subchondral bone through the cutting flute of the drill bit. (B) Illustration of a standard twist drill bit with the specific helix angle and flute design. (C) The tip of the drill bit features a specific point angle, flank and cutting edge. (D) Subchondral drilling with a Kirschner wire. The bone around the drill hole is compacted during the drilling procedure between the Kirschner wire and the subchondral bone. (E) Major types of Kirschner wire tip, including smooth trocar, smooth diamond, threaded trocar and threaded diamond. (F) Illustration of the four major types of Kirschner wire tip and the corresponding point geometry (bottom view).

Subchondral drilling, proposed for the treatment of osteochondritis dissecans by Smillie in 1957 (Smillie, 1957) and osteoarthritis by Pridie in 1959 (Pridie and Gordon, 1959), is a widely used marrow stimulation technique for articular cartilage repair in the clinical setting. Here, the standard procedure is routinely performed with either a surgical twist drill bit or a Kirschner wire to introduce several holes of a defined circular cross-section in the subchondral bone plate (Pridie and Gordon, 1959) (Fig. 1). Following a meticulous removal of the calcified cartilage layer, the exposed subchondral bone plate on the base of the defect is penetrated by the custom-made cutting tip of the instrument to a certain depth at a high speed of approximately 10,000 to 400,000 rpm (Fincham and Jaeblon, 2011). In preclinical studies involving small animals such as rats or rabbits, the drilling may be also performed manually (Shapiro et al., 1993; Song et al., 2014). The perforation of the subchondral medullary cavity releases bone marrow elements, which include stem cells and growth factors, into the base of the defect (Orth et al., 2013a). This bone marrow forms a blood clot and generates a repair tissue consisting of fibrocartilage, which is structurally and biomechanically inferior to the adjacent hyaline articular cartilage (Steinwachs et al., 2008).

During the past decade, the scientific interest has shifted away from viewing articular cartilage in isolation and placed more focus on the entire osteochondral unit, involving both articular cartilage and subchondral bone (Goebel et al., 2015; Hoemann et al., 2012; Madry, 2010). Advancements in radiographic analyses using high-resolution microcomputed tomography (micro-CT) systems allowed new insights into the microarchitecture of the subchondral bone to investigate the structural effects following cartilage repair treatment (Madry et al., 2010, 2016b; Menetrey et al., 2010). Combined with new histological findings and expansion of preclinical studies, the latest evidence outlines the significant shortcomings of the standard marrow stimulation techniques, including subchondral drilling (Chen et al., 2011a, 2009; Orth et al., 2012a, 2015). Among these shortcomings, some technical issues such as instrument type and design may partially explain the decline of the clinical outcome after about 2 years of the marrow stimulation treatment (Murawski et al., 2010).

Preclinical translational experimentations play a crucial role in cartilage research aimed at improving human healthcare. Systematic reviews and meta-analyses of preclinical studies are useful in optimizing the design of both preclinical and clinical studies (de Vries et al., 2014; Guo et al., 2018; Hooijmans et al., 2014a), making significant contributions to healthcare and more transparent translational medicine. For articular cartilage repair, a systematic review of the existing preclinical evidence for subchondral drilling is still lacking, despite its frequent clinical application. It remains unclear if and how any variables and translational models may influence the efficacy of the subchondral drilling.

We therefore systematically reviewed the effect of subchondral drilling to repair knee cartilage injury in preclinical translational models, focusing on the technical aspects of this procedure. This approach allowed us to analyze the temporal effect of subchondral drilling in both articular cartilage and subchondral bone repair. We also assessed the risk of bias of these preclinical data through either publication bias or factors relating to experimental design.

## RESULTS

### Study selection and description

**Fig. 2.**
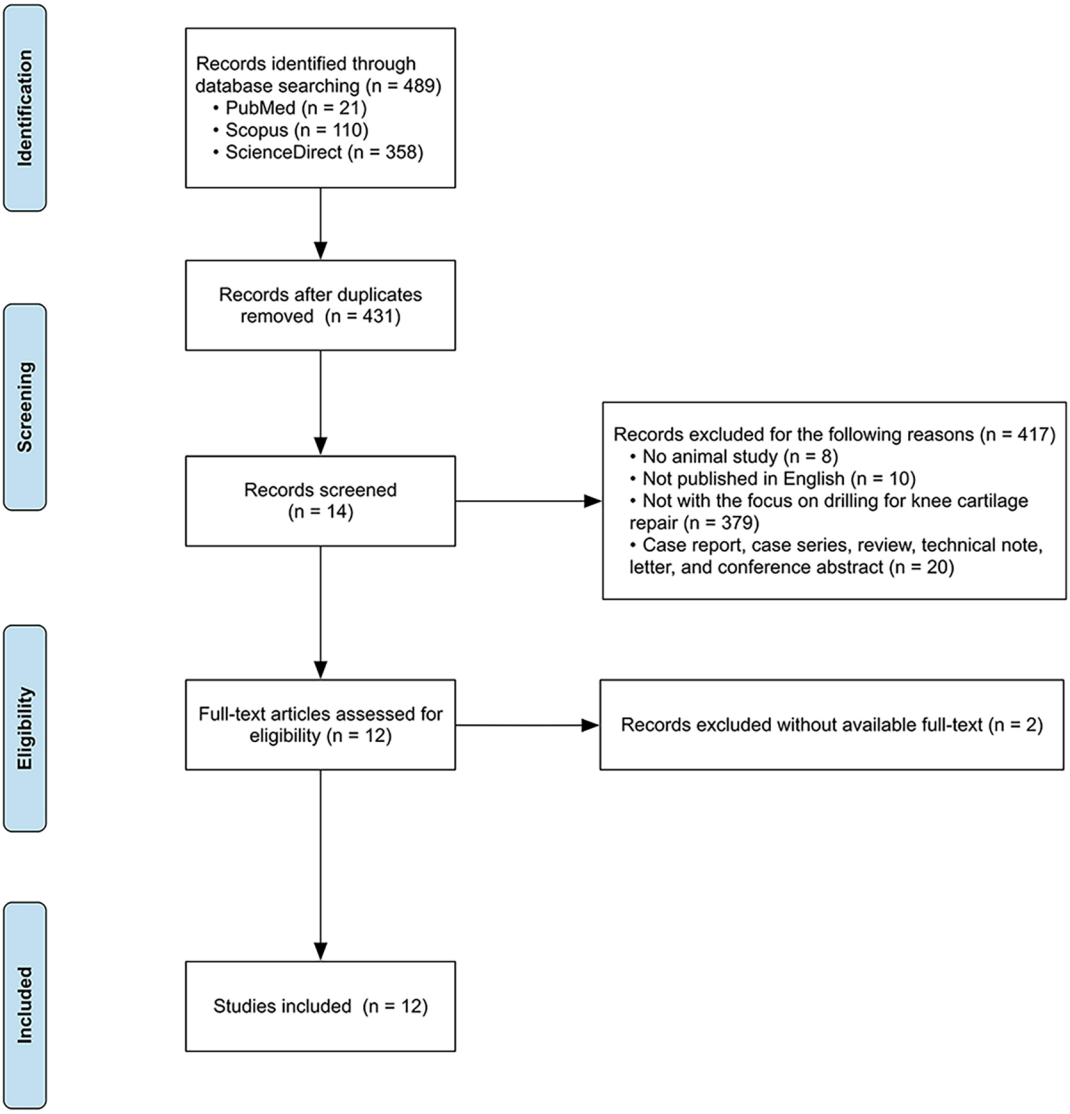
**Flow chart of study selection process.** The numbers of studies in each phase are shown in brackets.

The electronic search strategy (Tables S1-S3; until January 15, 2018) retrieved 21 records from PubMed/Medline, 110 from Scopus and 358 from ScienceDirect (Fig. 2). After duplicate removal, 431 references were screened based on title and abstract using DistillerSR. According to the predefined criteria, 417 reports were excluded and the remaining 14 reports were retrieved for full-text screening. In this phase, two studies were excluded as the full text was unavailable. Finally, the remaining 12 articles were identified for the quality assessment and the risk of bias evaluation.

The study characteristics involving both small and large animals were extracted and are summarized in Tables 1 and 2, respectively. Out of the 12 included studies, 6 studies were performed in rabbits (Chen et al., 2013a,b, 2011a,b, 2009; Menche et al., 1996) (50%), 4 in sheep (Eldracher et al., 2014; Giordano et al., 2011; Orth et al., 2012c, 2013b) (34%), 1 in goats (Lind et al., 2008) (8%) and 1 in rats (Hamanishi et al., 2013) (8%). Female animals were used in all studies in which animal gender was reported [4 studies did not report the animal gender (Giordano et al., 2011; Hamanishi et al., 2013; Lind et al., 2008; Menche et al., 1996)]. Rectangular defects were created in 9 studies (Chen et al., 2013a,b; Chen et al., 2011a,b, 2009; Eldracher et al., 2014; Menche et al., 1996; Orth et al., 2012c, 2013b) (75%) and cylindrical defects in 3 studies (Giordano et al., 2011; Hamanishi et al., 2013; Lind et al., 2008) (25%).

**Table 1.**
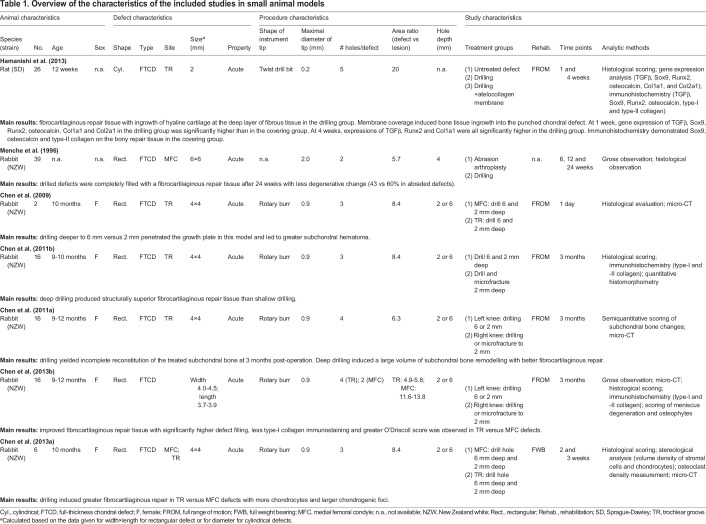
**Overview of the characteristics of the included studies in small animal models**

**Table 2.**
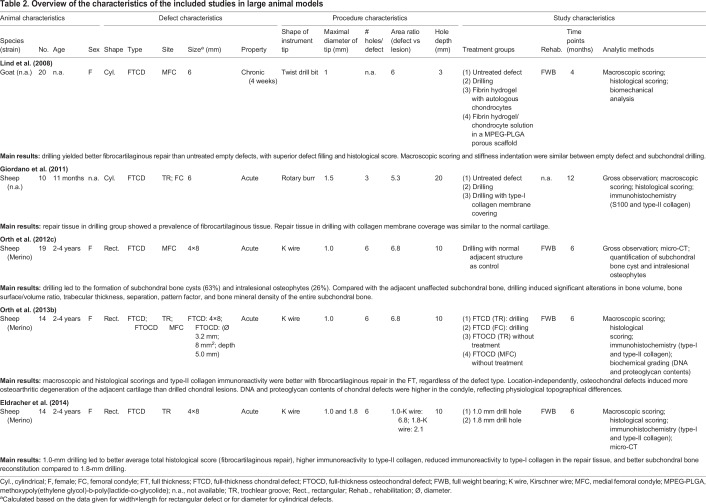
**Overview of the characteristics of the included studies in large animal models**

Subchondral drilling was performed with a surgical drill bit in 2 studies (Hamanishi et al., 2013; Lind et al., 2008) (17%), a Kirschner wire in 3 studies (Eldracher et al., 2014; Orth et al., 2012c, 2013b) (25%) and a rotary burr in 6 studies (Chen et al., 2013a, 2011a,b, 2009; Giordano et al., 2011) (50%). One study did not report the applied instrument type in the treatment (Menche et al., 1996). All studies reported the maximal diameter of the specific drill bit or Kirschner wire, but no further information about the instrument was provided in any papers, such as the point angle of the twist drill bit or the specific type of Kirschner wire (smooth or threaded; trocar or diamond). Defects in 11 studies (Chen et al., 2013a,b, 2011a,b, 2009; Eldracher et al., 2014; Giordano et al., 2011; Hamanishi et al., 2013; Menche et al., 1996; Orth et al., 2012c) were treated immediately after the defect creation (92%). Only the study from Lind et al. focused on the articular cartilage repair for chronic defects created 4 weeks before the time of the drilling treatment (Lind et al., 2008).

The average diameter of the instrument of drilling was 1.1±0.5 mm (mean±s.d.). In small preclinical models, the smallest instrument used in the rat (Hamanishi et al., 2013) was 0.2 mm and the largest one was applied in the rabbit (Menche et al., 1996) (2.0 mm). In large preclinical models, both the small and large instruments were used in sheep (Eldracher et al., 2014) (1.0 mm and 1.8 mm, respectively).

The area ratio was calculated individually for each included study as the ratio of the area of the cartilage defect (circular or rectangular) to the entire area of lesion(s)/hole(s) in the top view, representing the extent of subsequent subchondral impairment during the drilling process. The average area ratio was 7.7±4.4 and varied from 2.1 (Eldracher et al., 2014) to 20.0 (Hamanishi et al., 2013). This read to a percentage between the area of lesions/holes and the area of the entire defect between 5.0% and 47.6%. The average area ratio in small animal models was 1.7-fold of that in large animal models (9.5 and 5.6, respectively).

Eleven studies (92%) reported the depth of the drill hole, which varied in small animal models from 2.0 mm (Chen et al., 2013a,b; Chen et al., 2011a,b, 2009) to 6.0 mm (Chen et al., 2013a,b; Chen et al., 2011a,b, 2009) and in large animal models from 3.0 mm (Lind et al., 2008) to 20.0 mm (Giordano et al., 2011). One study in rats did not describe the depth of drill holes (Hamanishi et al., 2013).

All studies included full-thickness chondral defects located either in the trochlear groove or in the medial femoral condyle. Of note, only 3 studies included a control group of untreated full-thickness chondral defects (Giordano et al., 2011; Hamanishi et al., 2013; Lind et al., 2008).

Regarding the postoperative rehabilitation, no limb immobilization and exercise training was reported in any of the included studies. Immediate unrestricted activities were allowed in 5 studies (Chen et al., 2011a,b; Chen et al., 2013b, 2009; Hamanishi et al., 2013) (42%) and immediate full weight bearing was approved in 5 studies (Chen et al., 2013a; Eldracher et al., 2014; Lind et al., 2008; Orth et al., 2012c, 2013b) (42%). Two studies (Giordano et al., 2011; Menche et al., 1996) did not provide information on the postoperative animal management (16%).

Eight studies (67%) investigated a short-term outcome (<6 months) (Chen et al., 2013a,b; Chen et al., 2011a,b, 2009; Hamanishi et al., 2013; Lind et al., 2008; Menche et al., 1996) and the remaining 4 studies (33%) investigated the long-term outcome (≥6 months) (Eldracher et al., 2014; Giordano et al., 2011; Orth et al., 2012c, 2013b). Evaluation modalities of the outcome of articular cartilage repair involved gross observation [4 studies (Chen et al., 2013b; Giordano et al., 2011; Menche et al., 1996; Orth et al., 2012c)], macroscopic scoring [3 studies (Giordano et al., 2011; Lind et al., 2008; Orth et al., 2013b)], histological scoring [8 studies (Bonjar, 2013; Chen et al., 2013b, 2011b; Eldracher et al., 2014; Giordano et al., 2011; Hamanishi et al., 2013; Lind et al., 2008; Orth et al., 2013b)] and immunohistochemistry [6 studies (Chen et al., 2013b, 2011b; Eldracher et al., 2014; Giordano et al., 2011; Hamanishi et al., 2013; Orth et al., 2013b)]. Interestingly, although directly involved in the procedure, the subchondral bone reconstruction following the drilling treatment was only assessed in 6 studies (50%), using either semiquantitative scoring (Chen et al., 2013b), histomorphometric quantification of subchondral bone alterations (Orth et al., 2012c) or quantitative assessments using micro-CT (Chen et al., 2013a,b; Chen et al., 2011a,b, 2009; Eldracher et al., 2014). Biomechanical testing of the repair tissue was only performed in 1 study (Lind et al., 2008).

### Quality of reporting and risk of bias

**Fig. 3.**
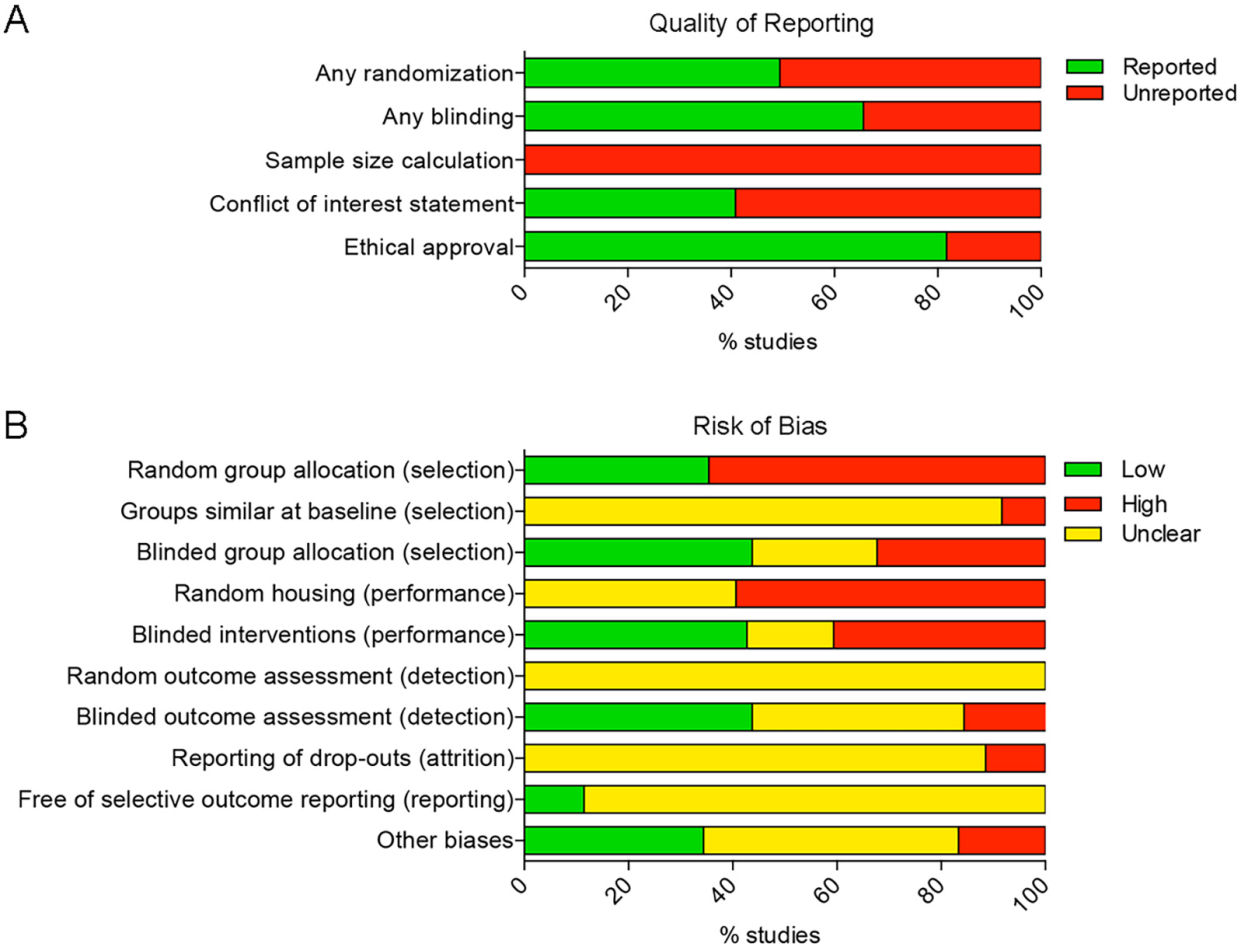
**Quality of reporting and risk of bias assessment using SYRCLE's risk of bias tool.** (A) Indicator of quality of reporting included randomization, blinding, sample size calculation, conflict of interest statement and ethical approval. (B) The risk of selection, performance, detection, attrition and other biases was assessed. Lack of (adequate) reporting of measurement to reduce bias resulted in a high percentage of unclear risk of bias for most items.

In the quality assessment of the included studies, randomization, blinding, sample size calculation, conflict of interest statement and ethical approval are key evaluation indices (Fig. 3A; Table 3). The score ‘yes’ (Y) indicates ‘reported’ and ‘no’ (N) indicates ‘unreported’. Randomization and blinding are essential to reduce bias, but were infrequently reported in the included studies. Out of the 12 studies included, 8 studies (Chen et al., 2013a,b; Chen et al., 2011a,b; Eldracher et al., 2014; Lind et al., 2008; Menche et al., 1996; Orth et al., 2013b) reported the blinding of the group allocation or outcome assessment, and 2 studies (Chen et al., 2013b; Orth et al., 2013b) described only the blinding of the histological assessment. None of the studies reported on a sample size calculation for the analysis of statistical power (Orth et al., 2013c).

**Table 3.**
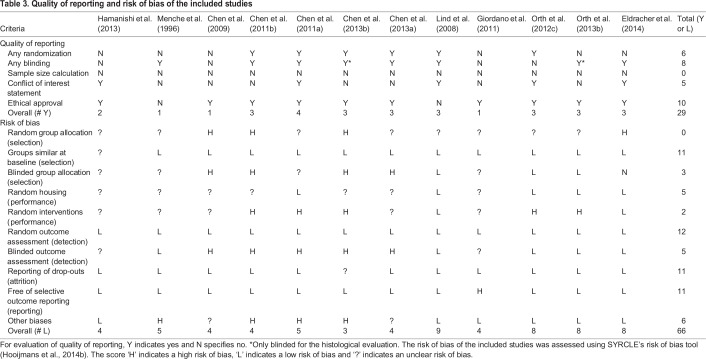
**Quality of reporting and risk of bias of the included studies**

The risk of bias of the included studies was assessed using SYRCLE's risk of bias tool, including 10 items related to 6 types of bias (selection, performance, detection, attrition, reporting and other biases) (Hooijmans et al., 2014b). The score ‘H’ indicates a high risk of bias, ‘L’ indicates a low risk of bias, and ‘?’ indicates an unclear risk of bias (Table S4). On average, the included studies reported 6 out of 10 characteristics (55±21%) (Fig. 3B). The lowest score was 3 out of 10 items (30%) and the highest scoring studies reported 9 items out of 10 (90%). However, none of the included studies reported randomization of the animals across treatment groups. Out of the 12 studies in which outcome of cartilage repair was evaluated by histological observation or scoring, only 42% reported blinding of the outcome assessment.

### Preclinical evidence of a temporal osteochondral repair pattern following subchondral drilling

#### Temporal cartilage repair pattern following subchondral drilling

The outcome of articular cartilage repair was assessed at specific time points varying from 1 week (Hamanishi et al., 2013) to 1 year (Giordano et al., 2011) in both small and large animal models.

##### Rat

In a rat model, Hamanishi et al. compared the 1-week and 4-week outcome of articular cartilage repair between untreated defects, drilled defects, and drilled defects with coverage of an atelocollagen membrane (Hamanishi et al., 2013). At 1 week, blood clots were present in the chondral defects of both the drilling and the membrane groups, while the cartilage defect in the untreated group appeared to be empty. At 4 weeks, all groups showed ingrowth of repair tissue into the defects, and the margins of the defects of drilling and untreated groups were clearly recognizable. The repair tissue in the drilled defects was smoother than that in the untreated defects, appearing like the normal surface of joint cartilage. Histological analysis at 4 weeks showed fibrous tissue filled within the untreated defects without staining with Safranin O. In the drilling group, the ingrowth of hyaline cartilage appeared in the deep layer of fibrous tissue in the defect. The histological score in the drilling group was significantly better than that in the untreated group.

Gene expression analysis was performed at 1 week and 4 weeks following the drilling procedure. At 4 weeks, TGFβ (1.6-fold higher at 1 than 4 weeks), Sox9 (13.9-fold), RUNX2 (1.4-fold), osteocalcin (5.5-fold), Col1al (2.9-fold) and Col2al (2.9-fold) were all decreased compared with the results of 1 week.

Immunohistochemical analysis of untreated defects at 4 weeks showed no immunoreactivity for TGFβ, Runx2, Col1a1 and Col2a1 at 4 weeks, whereas immunoreactivity of Sox9 was present in the deep layers of the fibrous repair tissue and that of osteocalcin in a shallow layer of the repair tissue. In the drilling group, TGFβ immunoreactivity was present in the deep layer of fibrous repair tissue above the area of ingrowth of hyaline cartilage. Also, Sox9- and Runx2-immunopositive cells were observed in the area of hyaline cartilage ingrowth in the drilling group, together with osteocalcin. Interestingly, positive immunoreactivity for type-I collagen was only observed in the fibrous repair tissue. In contrast, positive immunoreactivity for type-II collagen was observed in areas of hyaline cartilage ingrowth in the drilling group.

##### Rabbit

In a series of rabbit studies performed by Chen et al., full-thickness chondral defects located in the trochlea were treated by drilling with a 0.9 mm diameter drill bit (Chen et al., 2013a,b; Chen et al., 2011a,b, 2009). Stromal cell density in the drilled holes was found to be independent of days of repair (14 or 21 days) (Chen et al., 2013a).

At 2 weeks post-drilling, very few chondrocytes were seen in the drilled defects, but a significantly higher volume fraction of chondrocytes appeared within defects at 3 weeks postoperatively. These chondrocytes appeared deeper in the repairing holes above woven bone with more chondrogenic foci in the deep zone in surgical holes (Hamanishi et al., 2013). Both histology and micro-CT images showed new woven bone spicules at the base of the drilled holes at both 2 and 3 weeks. Drilling also elicited tartrate-resistant acid phosphatase (TRAP)-positive osteoclasts in remodelling woven bone, mainly at the base of holes at 14 and 21 days post-operation.

At 3 months post-drilling, trochlear defects were fully filled with repair tissue (Chen et al., 2013b). The superficial repair tissue mostly comprised fibrocartilage tissue with diminished intensity or depleted Safranin-O staining (Chen et al., 2011a). Extensive Safranin-O staining of proteoglycan-rich repair tissue was observed mostly in the bottom region of the repair tissue. Positive immunoreactivity for type-II collagen was more widespread in the repair matrix. The tidemark and zonal organization of articular cartilage were not reestablished. Confirmed bonding between repair tissue and adjacent cartilage was frequently not achieved. Basal attachment of the repair tissue to the underlying bone was observed in 80% of defects.

##### Goat

In an attempt to mimic the clinical situation of chronic cartilage disturbances, Lind et al. evaluated the 4-month articular cartilage repair of the chronic defects in the medical femoral condyle (Lind et al., 2008). Those defects were created 4 weeks prior to the drilling treatment. At 4 months after drilling, no difference of the macroscopic International Cartilage Regeneration & Joint Preservation Society (ICRS) score was observed between the drilled and untreated defects. The drilled defects yielded a considerably higher percentage of defect filling (30.5% vs 12.5%) and an improved overall O'Driscoll histological score value (11.5 vs 3.0) compared with the untreated defects. In the region of subchondral bone without coverage of repair tissue in untreated defects, no histological signs of bone destruction were observed. Mechanical testing revealed no difference between drilled and untreated defects, and the repair tissue in both drilled and untreated defects was significantly stiffer than the normal cartilage.

##### Sheep

A series of studies investigated the 6-month outcome of the subchondral drilling using a Kirschner wire in sheep (Eldracher et al., 2014; Orth et al., 2012c, 2013b). At 6 months after drilling, cartilage repair and osteoarthritis changes were evaluated by macroscopic, histological, immunohistochemical and biochemical methods. No joint effusion, macroscopic inflammation, periarticular osteophytes or adhesions were detected. Most defects were insufficiently filled with repair tissue, with the colour varying between translucent and white (Orth et al., 2012c). Compared with the defects in the femoral condyle, the trochlear defects yielded a better articular cartilage repair, as shown by the improved macroscopic and histological score and the enhanced immunoreactivity to type-II collagen (Orth et al., 2013b). When compared with large drill holes (1.8 mm in diameter), small drill holes (1.0 mm) led to significantly improved histological matrix staining, cellular morphological characteristics, average total histological score, higher immunoreactivity to type-II collagen and reduced immunoreactivity to type-I collagen in the repair tissue. No significant differences were observed regarding the osteoarthritic changes in the cartilage adjacent to the defects between the 1.0 mm and 1.8 mm drilling groups (Eldracher et al., 2014).

Another long-term follow-up in the sheep model from Giordano et al. applied a 1.5 mm drill bit to drill to 20 mm (Giordano et al., 2011). At 12 months after treatment, untreated defects were characterized by an incomplete filling. The drilled defects showed a complete filling of the lesions. The repair tissue was smooth, level with normal adjacent normal cartilage and similar in colour and thickness. Histological examination showed that the repair tissue within both untreated and drilled defects was characterized by fibrous repair with poor intensity of Safranin-O staining.

#### Temporal subchondral bone repair pattern following subchondral drilling

Interestingly, the repair of the subchondral bone has received little attention so far, although it is, by definition, not only involved in the drilling procedure but also changes its character following osteochondral repair.

##### Rabbit

Chen et al. reported differences in osteocyte necrosis and acute subchondral bone structure between subchondral drilling and microfracture in the rabbit model (Chen et al., 2009). Histological observation and micro-CT evaluation were performed 1 day following the drilling procedure. Notably, only 2 rabbits (4 holes for microfracture to 2 mm; 8 holes for drilling to 2 mm; 4 holes for drilling to 6 mm) were used in this study. In another study (Chen et al., 2011a), 5 variables of subchondral bone repair were semi-qualitatively assessed, including the presence of residual holes and cysts, bone overgrowth above the projected tidemark, bone resorption, integration to the lateral adjacent bone plate, and restoration of the subchondral bone plate. The data showed no significant difference regarding these variables between microfracture to 2 mm depth and drilling to either 2 mm or 6 mm depth.

##### Sheep

In the sheep model, a 6-month outcome of subchondral bone structure with micro-CT was reported (Orth et al., 2012c). The normal subchondral bone adjacent to the drilled defect served as control. Subchondral drilling led to the formation of subchondral bone cysts (63%) and intralesional osteophytes (26%). Compared with the adjacent unaffected subchondral bone, drilling also induced significant alterations in bone volume, bone surface/volume ratio, trabecular thickness, separation, pattern factor, and bone mineral density of the entire subchondral bone.

#### Specific aspects of modifiable variables for subchondral drilling and their effects on osteochondral repair

##### Effect of drill bit diameter on osteochondral repair

Eldracher et al. compared the 6-month outcome of the osteochondral repair in a Merino sheep model treated with a 1.0 mm or 1.8 mm drill bit (Eldracher et al., 2014). The outcome was evaluated with macroscopic scoring, histological scoring, immunohistochemistry (type-I and type-II collagen) and micro-CT. Drilling with a 1.0 mm drill bit led to a better average total histological score, higher immunoreactivity to type-II collagen, reduced immunoreactivity to type-I collagen in the repair tissue and better subchondral bone reconstitution compared with 1.8 mm drilling. The 1.8 mm drill led to a reduced bone mineral density of the peri-hole subchondral bone compared with adjacent subchondral bone. No significant differences of osteoarthritic changes in the adjacent cartilage were detected between treatment groups.

##### Effect of drilling depth on osteochondral repair

A series of studies were performed in the rabbit model to compare drilling to a depth of 6 mm or 2 mm (Chen et al., 2013a,b; Chen et al., 2011a,b, 2009). Drilling deeper, to 6 mm versus 2 mm, penetrated the epiphyseal scar in this model and led to greater subchondral hematoma (Chen et al., 2009), produced superior cartilage repair (Chen et al., 2011b), and induced a larger volume of the subchondral bone remodelling than shallower drilling (Chen et al., 2011a).

##### Topographic variation of osteochondral repair

In the rabbit model, Chen et al. observed a better cartilage repair outcome in defects located in the trochlea compared with defects in the medial femoral condyle (Chen et al., 2013b). The same group also reported that drilling in trochlear defects induced greater chondrogenesis than defects in the medial femoral condyle, with more chondrocytes and larger chondrogenic foci (Chen et al., 2013a).

A similar topographic variation of osteochondral repair was reported in the sheep study by Orth et al. (2013b). The 6-month outcome of articular cartilage repair of full-thickness chondral defects in both trochlea and medical femoral condyle was evaluated after subchondral drilling applying a 1.0 mm Kirschner wire. Macroscopic and histological articular cartilage repair and type-II collagen immunoreactivity were improved in the femoral trochlea, regardless of the defect type. DNA and proteoglycan contents of chondral defects were higher in the condyle, reflecting physiological topographical differences. Independently of location, osteochondral defects induced more osteoarthritic degeneration of the adjacent cartilage than drilled chondral lesions.

## DISCUSSION

The standardization of the subchondral drilling technique and the selection of the appropriate preclinical model is crucial for comparing different translational studies and ultimately for the long-term success of this surgical technique. This systematic review summarizes the translational evidence and confirms the efficacy of subchondral drilling for articular cartilage repair in the knee joint. Subchondral drilling has been proven to be superior to non-treatment of defects in multiple translational models. The temporal patterns of both articular cartilage and subchondral bone repair following drilling are reviewed. Specific aspects of modifiable variables for subchondral drilling and their influences on articular repair are also examined.

Challenges remain to define the optimal technique of drilling. For the twist drill bit, factors such as drilling force, speed, length of the chisel edge, degree of the helical angle and the rake angle (the angle at which the cutting surface is presented to the materials) alter the effectiveness of bone drilling (Natali et al., 1996). Inherent characteristics, for instance, the design of the cutting tip, flute shape and manufacturing material, also influence the efficiency of drilling (Jacob et al., 1976). Another unresolved problem for drilling is the geometrical inaccuracy in hole size and location. This frequently occurs due to the undesired spiralling and slipping of the instrument on the bone surface and within the subarticular spongiosa (Isbilir and Ghassemieh, 2013). Previous studies showed that surface properties, bone contour and the drilling angle might affect the targeting of the drill bit (Saha et al., 1982). In contrast to drill bits, several characteristics of the tip of a Kirschner wire also contribute to penetration efficiency, including the tip configuration (diamond or trocar), rake angle and drill speed (Graebe et al., 1992; Namba et al., 1987). Therefore, in either instrument design or application, one might need to take all relative items of the instrument into account to ensure effective and targeted drilling.

The configuration of the surgical instrument, either twist bit or Kirschner wire, greatly affects the structure of the treated subchondral bone, which may alter the final outcome of osteochondral repair (Orth et al., 2012b). The different pattern of subchondral bone structure between subchondral drilling and microfracture was reported in rabbits (Chen et al., 2009). The data from the present study shows that neither of these 2 studies explained the reason for the selection of the specific instrument nor provided detailed description about the instrument tip, such as the point angle, the flute design or the Kirschner wire category (trocar or diamond; smooth or threaded). When selecting sizes and qualities of a tool for drilling in an animal model, parameters such as the thickness of both the articular cartilage and the subchondral bone plate, together with the degree of mineralization and mechanical stiffness of the subchondral bone plate, relation of the size of the defect with the mean condylar or trochlear width (area/width index) or animal weight have to be considered (Orth et al., 2012c). Such heterogeneity of the drilling technique may partially account for the inter-study variation of the outcome in different animals. Therefore, additional explorations about the relationship between instrument design and osteochondral repair are necessitated to guarantee an optimal long-term success of cartilage repair. Also, in some reports the drilling was performed through articular cartilage at the base of the defect (Shamis et al., 1989; Vachon et al., 1986) – this has to be avoided because, in a clinical situation, all cartilage, including the calcified layer, is always removed and the drilling is performed through the exposed subchondral bone (Frisbie et al., 2006b).

Interestingly, for clinical articular cartilage repair, the most common instruments for subchondral drilling are a twist drill bit and a Kirschner wire. However, in half of the included studies (6 of 12 studies), a rotary burr was applied for subchondral drilling in rabbits (Chen et al., 2013a,b; Chen et al., 2011a,b, 2009) and sheep (Giordano et al., 2011). Such rotary burrs are mainly used in neurosurgery (Williams et al., 2001) and dental surgery (Bonjar, 2013). In the series of rabbit studies, a different structure of the treated subchondral bone was observed between microfracture and drilling, and a significantly better cartilage repair was reported for deep drilling (6.0 mm) than for shallow drilling (2.0 mm), using such a rotary burr. Considering the variation of the instrument and the difficulty in producing a standardized cylindrical hole using such a hand-held instrument with a round or oval tip, it is unclear whether similar results can be expected in patients who are treated with a twist drill bit or a Kirschner wire (Van der Worp et al., 2010). More preclinical and clinical studies with such instruments (drill bit or Kirschner wire) will further substantiate the effects on the subchondral bone.

As a by-product of the drilling process, the possibility of thermal osteonecrosis is a concern when applying subchondral drilling during either open arthrotomy or dry arthroscopy (Alam et al., 2015). Farhan-Alanie and Hall showed temperature changes and chondrocyte death during drilling in a bovine metatarsophageal joint and demonstrated that application of a modified irrigation solution enabled chondroprotection (Farhan-Alanie and Hall, 2014). In this systematic review, only 5 studies described the heat protection step with constant irrigation with precooled sterile Ringer lactate solution (Chen et al., 2013a,b; Chen et al., 2011a,b, 2009). Moreover, a newly designed drill bit with an internal cooling system might provide a more targeted and effective method to deliver the irrigation fluid (Augustin et al., 2012). Therefore, applying such an irrigation procedure in these cases is recommended, and its use needs to be described.

Abrasion arthroplasty, subchondral drilling and microfracture are all considered as marrow stimulation techniques, where the chondral lesion gains access to reparative elements from the bone cavity through the openings of the subchondral bone plate. Abrasion arthroplasty was ever popular in the 1980s and gradually fell into disfavour (Johnson, 1986). Remarkably, the superiority of subchondral drilling over abrasion arthroplasty for articular cartilage repair has only been shown in the rabbit model, but with a poor quality of reporting and a high risk of bias (Menche et al., 1996). To the best of our knowledge, no robust and comprehensive comparison has ever been undertaken between abrasion arthroplasty and subchondral drilling for clinical cartilage repair (Bert, 1997). Similar debates also remain for the superiority between traditional subchondral drilling and microfracture in both preclinical (Chen et al., 2013a, 2011b) and clinical (Hunziker, 2002; Steadman et al., 1997) settings. Therefore, additional research is still required to determine whether any one of the three techniques is superior to the others.

The innate inter-joint difference of the structure, composition and biomechanics has been revealed in the literature (Eger et al., 2002; Fetter et al., 2006). Such variation may partially explain the different pattern of cartilage repair between the knee and ankle. In the knee, smaller and deeper subchondral drill holes are better to improve marrow stimulation of articular cartilage defects (Chen et al., 2011b; Eldracher et al., 2014). However, repair pattern is different in the ankle. In the sheep study from Kok et al., when talar osteochondral defects were drilled with either six 0.45 mm holes to 2 mm and 4 mm depth or three 1.1 mm holes to 3 mm depth, no differences in cartilage healing were detected at 24 weeks postoperatively despite the different geometry of the microfracture holes (Kok et al., 2013).

*In vivo* preclinical studies are required for testing of cartilage reconstruction techniques and novel therapeutic biomaterials. Compared with small animals such as rats or rabbits, the stifle joint of large animal models such as sheep or horses provides a better approximation to the human knee in terms of articular cartilage thickness and joint size (Frisbie et al., 2006a). Considering economic feasibility and practicality, it is generally widely accepted to use a small animal model for the initial investigation and a large animal model for the final preclinical evaluation before beginning a clinical trial (Moran et al., 2016). Moreover, the interspecies dissimilarity among different animal models should be recognized. For certainty, one finding from one animal model (or species) may still need to be confirmed in another animal model (or species). Finally, systematic analysis of the existing preclinical evidence performed before the start of a clinical trial may save resources, improve the safety of participants in clinical trials and ultimately ensure a better healthcare (Ritskes-Hoitinga et al., 2014).

Although chronic and sometimes degenerative chondral lesions are much more common in the clinical setting (Madry et al., 2016a), this fact is not adequately reflected in the preclinical studies examined here. Often, an acute defect was created and treated immediately. This may be explained in part by the restrictive regulations in animal experimentations. Degenerative changes of the chronic chondral lesions are characterized with eburnated bone, bony sclerosis and thickening of the subchondral bone plate (Johnson, 1990). In a drilling procedure performed in such a defect, it is more difficult to establish a stable perpendicular rim within the softened adjacent cartilage, possibly making a secure fixation of the bone marrow clot more difficult. In 4-week-old chronic defects of goats, sclerosis was observed in the repair tissue from either drilled or undrilled defects as shown by a higher stiffness than the normal cartilage (Lind et al., 2008). However, subchondral drilling still generated increased defect filling and improved histological score compared to the untreated defects. No histological signs of bone destruction were observed in the denuded subchondral bone. These data might imply that subchondral drilling is also effective in chronic defects, although more in-depth and long-term preclinical and clinical studies are warranted.

Based on the current lacking translational evidence, future translational researches on subchondral drilling are therefore recommended, including comparisons between drill bits and Kirschner wires, re-evaluation of the outcomes obtained from previous studies using a rotary burr for subchondral drilling, standardization of drill design and outcome reporting, applying irrigation in an open drilling procedure to limit heat necrosis (and reporting this step in manuscripts), and performing adequate and appropriate rehabilitation to mimic the clinical process of treatment. Moreover, multiple risks of bias should be avoided in the design and implementation of translational studies. Selective and insufficient reporting should also be avoided.

In conclusion, this systematic review indicates that subchondral drilling yields improved short-term structural articular cartilage repair compared with spontaneous healing in multiple small and large animal models. However, specific aspects of modifiable variables still need to be taken into consideration for the design, modification and application of subchondral drilling. Moreover, the current preclinical studies on subchondral drilling may not be optimally designed and reported, and further optimization may be necessary for successful translation to the clinical setting.

## MATERIALS AND METHODS

### Search strategy

This systematic review is reported according to the Preferred Reporting Items for Systematic Reviews and Meta-Analyses (PRISMA) guideline (Moher et al., 2015). A pre-specified protocol was used for the study selection and extraction of study characteristics. Several systematic searches were conducted in the PubMed (1946-present), Scopus (1966-present) and ScienceDirect (1995-present) repositories, the latest on January 15, 2018, by using different synonyms, Medical Subject Headings (MeSH) and Emtree terms (for PubMed and Scopus, respectively) to cover any preclinical study of subchondral drilling (Tables S1-S3). To ensure a comprehensive search of preclinical studies, a specifically designed and validated search filter was used to search in the PubMed database (Hooijmans et al., 2010). The article language was restricted to English.

### Study selection

After removal of duplicates, selection of studies was performed using a web-based systematic review software (DistillerSR, Evidence Partners, Ottawa, Canada). All references were first screened for inclusion based on the title and abstract. The following inclusion criteria were applied: the study (1) is an original article presenting unique data with a control group in preclinical model of articular cartilage repair, (2) describes the procedures of cartilage defect creation and treatment, (3) investigates the technical aspects of subchondral drilling treatment, and (4) examines the outcomes of osteochondral repair. Subsequently, the full-text manuscripts of eligible studies were reviewed for inclusion. Studies focusing on the outcomes of subchondral drilling augmented with specific matrix, scaffolds, cells (stem cell or chondrocyte), cytokines and biological factors were excluded. In both phases, references were independently assessed for inclusion by two researchers (L.G. and L.K.H.G). In case of discrepancies, consensus was obtained through discussion with the senior researcher (H.M.).

### Data extraction

The following items were extracted from each included study using DistillerSR, including reference details (publication year and name), animal features (species, number, age and sex), defect characteristics (shape, type, location and property), procedure details (instrument and intervention characteristics) and study design (treatment groups, rehabilitation, time points, analytic methods and main results). For each study, the value ratio between defect area and total lesion (hole) area was calculated accordingly.

### Methodological quality of studies

After reviewing the experimental details on animals, methods and materials, quality of data reporting was evaluated for randomization, blinding, sample size calculation, conflict of interest statement and ethical approval. For these items, a ‘yes’ indicates reported, and a ‘no’ indicates unreported.

The risk of bias of the included studies was assessed using SYRCLE's risk of bias tool (Hooijmans et al., 2014b). Ten items were reviewed regarding 6 types of bias (selection, performance, detection, attrition, reporting and other biases). The score ‘yes’ indicates a low risk of bias, ‘no’ indicates a high risk of bias and ‘?’ indicates an unclear risk of bias.

## Supplementary Material

Supplementary information
