# Supplementary material for: Subchondral drilling for articular cartilage repair: a systematic review of translational research
Source: Dis Model Mech. 2018 Jun 19;11(6):dmm034280. doi: 10.1242/dmm.034280 (PMC6031351; doi:10.1242/dmm.034280)
Supplement: Supplementary information [file dmm-11-034280-s1.pdf]

Several systematic searches were conducted in the PubMed version of MEDLINE, Scopus, and ScienceDirect, the latest on January 15, 2018, by using different synonyms, Medical Subject Headings (MeSH) and Emtree terms (for PubMed and Scopus, respectively) to cover any study regarding subchondral drilling. To ensure a comprehensive search of translational studies, a specifically designed and validated search filter was used for search in the PubMed database (Hooijmans et al., 2010).

Searching strategy in PubMed

Table S1. The search components and search terms used for literature searching in PubMed.

Search components	Search terms
Pridie drilling	Arthroscopic Subchondral Drilling [MeSH terms] OR Subchondral Drilling, Arthroscopic [MeSH terms] OR Subchondral Drillings, Arthroscopic [MeSH terms] OR Drilling [tiab] OR Drill [tiab] Subchondral perforation [tiab] OR Subchondral drilling [tiab]
Articular cartilage defect	Cartilage, Articular [MeSH terms] OR Articular Cartilage [MeSH terms] OR Cartilage defect [tiab] OR (cartilage [tiab] AND defect [tiab])
Animals	Laboratory animal search filter

The combined search terms were as follows: *(Arthroscopic Subchondral Drilling [MeSH terms] OR Subchondral Drilling, Arthroscopic [MeSH terms] OR Subchondral Drillings, Arthroscopic [MeSH terms] OR Drilling [tiab] OR Drill [tiab] Subchondral perforation [tiab] OR Subchondral drilling [tiab]) AND (Cartilage, Articular [MeSH terms] OR Articular Cartilage [MeSH terms] OR Cartilage defect [tiab] OR (cartilage [tiab] AND defect [tiab]))*

Thirty-eight records were identified through searching with the above search terms in

Disease Models & Mechanisms • Supplementary information

PubMed/Medline. The “Animal filter“ designed by Hooijmans *et al*. (Hooijmans et al., 2010) was utilized and finally retrieved 21 articles from Pubmed.

Literature searching in Scopus

Table S2. The search components and search terms used for literature searching in Scopus.

Search components	Search terms
Pridie drilling	ALL ("marrow stimulation") OR ALL ("Pridie Drilling") OR ALL ("Subchondral Drilling") OR ALL ("Drill") OR ALL ("subchondral perforation") OR ALL ("Drilling") AND TITLE-ABS-KEY("drill")
Articular cartilage defect	TITLE-ABS-KEY (“cartilage”)
Animals	TITLE-ABS-KEY (“animal” OR “translation*”)

The combined search terms were as follows: *ALL ("marrow stimulation") OR ALL ("Pridie drilling") OR ALL ("subchondral drilling") OR ALL ("drill") OR ALL ("subchondral perforation") OR ALL ("drilling") AND TITLE-ABS-KEY ("drill") AND TITLE-ABS-KEY ("cartilage") AND TITLE-ABS-KEY ("animal" OR "translation*")*

One hundred ten articles were identified from Scopus database.

Literature searching in ScienceDirect

Table S3. The search components and search terms used for literature searching in ScienceDirect.

Search components	Search terms
Pridie drilling	(marrow stimulation) OR (subchondral drilling) OR (Pridie drilling) OR (drilling) OR (drill) OR (subchondral perforation)
Articular cartilage defect	title-abs-key (cartilage)
Animals	title-abs-key ((animal) OR (translation*))

The combined search terms were as follows: *(marrow stimulation) OR (subchondral drilling) OR (Pridie drilling) OR (drilling) OR (drill) OR (subchondral perforation) AND title-abs-key (cartilage) AND title-abs-key ((animal) OR (translation*))*

Three hundred thirty-eight articles were identified from ScienceDirect database.

Table S4. The SYRCLE's tool for assessing risk of bias for animal studies (Hooijmans et al., 2014).

Item	Type of bias	Domain	Description of domain	Review authors judgment
1	Selection bias	Sequence generation	Describe the methods used, if any, to generate the allocation sequence in sufficient detail to allow an assessment whether it should produce comparable groups.	Was the allocation sequence adequately generated and applied? (*)
2	Selection bias	Baseline characteristics	Describe all the possible prognostic factors or animal characteristics, if any, that are compared in order to judge whether or not intervention and control groups were similar at the start of the experiment.	Were the groups similar at baseline or were they adjusted for confounders in the analysis?
3	Selection bias	Allocation concealment	Describe the method used to conceal the allocation sequence in sufficient detail to determine whether intervention allocations could have been foreseen before or during enrolment.	Was the allocation adequately concealed? (*)
4	Performance bias	Random housing	Describe all measures used, if any, to house the animals randomly within the animal room.	Were the animals randomly housed during the experiment?
5	Performance bias	Blinding	Describe all measures used, if any, to blind trial caregivers and researchers from knowing which intervention each animal received. Provide any information relating to whether the intended blinding was effective.	Were the caregivers and/or investigators blinded from knowledge which intervention each animal received during the experiment?
6	Detection bias	Random outcome assessment	Describe whether or not animals were selected at random for outcome assessment, and which methods to select the animals, if any, were used.	Were animals selected at random for outcome assessment?
7	Detection bias	Blinding	Describe all measures used, if any, to blind outcome assessors from knowing which intervention each animal received. Provide any information relating to whether the intended blinding was effective.	Was the outcome assessor blinded?
8	Attrition bias	Incomplete outcome data	Describe the completeness of outcome data for each main outcome, including attrition and exclusions from the analysis. State whether attrition and exclusions were reported, the numbers in each intervention group (compared with total randomized animals), reasons for attrition or exclusions, and any re-inclusions in analyses for the review.	Were incomplete outcome data adequately addressed? (*)
9	Reporting bias	Selective outcome reporting	State how selective outcome reporting was examined and what was found.	Are reports of the study free of selective outcome reporting? (*)
10	Other	Other sources of bias	State any important concerns about bias not covered by other domains in the tool.	Was the study apparently free of other problems that could result in high risk of bias? (*)

*Items in agreement with the items in the Cochrane Risk of Bias tool. For each item, the score "H" indicates a high risk of bias, "L" indicates a low risk of bias, and "?" indicates an unclear risk of bias.

Disease Models & Mechanisms • Supplementary information

References

Hooijmans, C. R., Rovers, M. M., de Vries, R. B., Leenaars, M., Ritskes-Hoitinga, M. and Langendam, M. W. (2014). SYRCLE's risk of bias tool for animal studies. *BMC medical research methodology* **14**, 43.

Hooijmans, C. R., Tillema, A., Leenaars, M. and Ritskes-Hoitinga, M. (2010). Enhancing search efficiency by means of a search filter for finding all studies on animal experimentation in PubMed. *Laboratory animals* **44**, 170-175.

Disease Models & Mechanisms • Supplementary information
